# Severe maternal morbidity and maternal near miss in the extremes of reproductive age: results from a national cross- sectional multicenter study

**DOI:** 10.1186/1471-2393-14-77

**Published:** 2014-02-20

**Authors:** Fernando César Oliveira, Fernanda Garanhani Surita, João Luiz Pinto e Silva, José Guilherme Cecatti, Mary Angela Parpinelli, Samira M Haddad, Maria Laura Costa, Rodolfo Carvalho Pacagnella, Maria Helena Sousa, João Paulo Souza

**Affiliations:** 1 Department of Obstetrics and Gynecology, University of Campinas, Rua Alexander Fleming, 101, 13083-881 Campinas, SP, Brazil; 2 Department of Gynecology and Obstetrics, Federal University of Paraná, Curitiba, PR, Brazil; 3 Cemicamp: Centre for Research on Reproductive Health of Campinas, Campinas, Brazil

**Keywords:** Maternal near-miss, Obstetric complication, Maternal death, Maternal age, Extremes of reproductive age, Maternal morbidity

## Abstract

**Background:**

The aim of this study was to assess severe maternal morbidity (SMM) and near miss (NM) cases among adolescent girls and women over 35 years of age in the Brazilian Network for Surveillance of Severe Maternal Morbidity, using a set of standard criteria, compared to pregnant women aged 20 to 34 years.

**Methods:**

A cross-sectional multicenter study conducted in 27 referral obstetric units in Brazil. All pregnant women admitted to these centers during a one-year period of prospective surveillance were screened to identify cases of maternal death (MD), NM and other SMM*.* Indicators of maternal morbidity and mortality were evaluated for the three age groups. Sociodemographic, clinical and obstetric characteristics, gestational and perinatal outcomes, main causes of morbidity and delays in care were also compared. Two multiple analysis models were performed, to estimate the adjusted prevalence ratio for identified factors that were independently associated with the occurrence of severe maternal outcome (SMO = MNM + MD).

**Results:**

Among SMM and MD cases identified, the proportion of adolescent girls and older women were 17% each. The risk of MNM or death was 25% higher among older women. Maternal near miss ratio and maternal mortality ratios increased with age, but these ratios were also higher among adolescents aged 10 to 14, although the absolute numbers were low. On multivariate analysis, younger age was not identified as an independent risk factor for SMO, while this was true for older age (PR 1.25; 1.07-1.45).

**Conclusions:**

SMO was high among women below 14 years of age and increased with age in Brazilian pregnant women.

## Background

Maternal mortality is a key heath care indicator related to a country’s level of development. Adolescent girls are most vulnerable to social maladjustment. This may be the cause or result of unexpected pregnancies, which increase the occurrence of unsafe abortions, the discontinuation of formal education and are associated with lower adherence to prenatal care [1]. In a similar fashion, although for distinct reasons, older pregnant women are also considered at high risk of obstetric complication due to a higher prevalence of associated morbid conditions and/or multiparity [2,3].

The study of maternal death (MD) is fraught with difficulties and therefore, in the past years, women who survive severe conditions of pregnancy or near-miss (NM) complications have attracted great interest as a source of information on processes that can lead to death. The investigation of these cases can be used as a complementary method to audits and inquiries into MD [4,5]. The investigation of NM events provides important details on factors that may contribute to both MD and NM. Knowledge on the prevalence and causes of NM may indeed constitute a new form of evaluating obstetric care [6,7].

In 2009, the World Health Organization (WHO) defined Maternal Near-Miss as a woman who almost died but survived a complication that occurred during pregnancy, childbirth or within the 42 days following pregnancy termination. Standardized criteria for NM identification have been defined, according to organ dysfunction and/or failure- and the WHO recommends that this approach should be used to assess quality of obstetric care [6,8,9]. Clinical signs, laboratory tests and management interventions were used, aimed at diagnosing organ dysfunction or failure. These criteria were previously validated in a Brazilian obstetric population [10]. However, in the published literature a wide variety of criteria used to identify cases of maternal NM still prevail [11].

Although pregnancy during adolescence is a constant public health issue, due to the recognized higher risk for the mother and infant, in addition to the strong biological, psychological and social impact, maternal death associated with adolescence is more well-known than the corresponding morbidity. The risk of maternal death for women aged 15 to 19 years is twice the risk for women aged 20 to 24 years. For those aged 10 to 15 years, the risk of death may be even five-fold higher, compared to women aged 20 to 24 years [12]. Half of all adolescent births in the world occur in only seven countries: Bangladesh, Brazil, Democratic Republic of Congo, Ethiopia, India, Nigeria and the United States [13]. Apart from the United States, the remaining six countries still have a high maternal mortality ratio. Furthermore, pregnancies among women aged over 35 years are increasing worldwide. In Finland, the rate of old age pregnancy increased from a total of 16.7% in 1997 to 19.2% in 2007 [14]. Data from the WHO for developing countries around the world reveal a prevalence of 10.6% of pregnancies in women aged over 35 years between 2004 and 2008 [15]. Pregnancies in older women are also associated with a higher maternal and perinatal morbidity and mortality, with a greater risk of various morbid conditions, such as hypertension and diabetes, anomalous presentation, intrapartum fetal distress, cesarean delivery and postpartum hemorrhage. In older pregnant women following delivery, Apgar scores were lower and there was a higher incidence of low birth weight and preterm when compared to younger women [2,3,16,17].

However, information on the occurrence of near-miss and severe maternal morbidity in both extremes of reproductive age is scarce. The lack of knowledge considering the impact of the problem and the existence of any associated factors liable to intervention makes it difficult to improve healthcare and prevent complications in these age groups. Thus, the aim of the current study is to explore the characteristics of severe maternal morbidity, with special focus on the extremes of reproductive age, among women identified in the Brazilian Network for Surveillance of Severe Maternal Morbidity. Knowledge of these features may lead to improvement in public policies and result in better care of women developing severe maternal morbidity in the extremes of reproductive ages.

## Methods

Detailed information on the study’s method and procedures were previously published [5,18]. Briefly, this was a multicenter cross-sectional study, implemented in 27 referral obstetric units in all geographical regions in Brazil. From July 2009 to June 2010, a prospective surveillance was carried out and data was collected to identify cases of potentially life-threatening maternal conditions (PLTC), maternal near-miss and maternal death, using the new WHO’s concept and criteria [8]. The present article refers to an analysis focused on the occurrence of severe maternal morbidity (SMM = PLTC + MNM + MD) and severe maternal outcome (SMO = MNM + MD) related to the extremes of maternal age.

Sample size calculation determined that about 75,000 deliveries should be surveyed to identify around 750 near-miss cases, using an approximate theoretical incidence of 10 near miss cases per 1000 deliveries as basis for calculation [6].

Maternal age was considered an independent variable that was categorized into three groups: adolescence (between 10 and 19 years, with a subgroup aged 10-14 years and another aged 15-19 years), pregnant women over age 35 (with a subgroup aged 35-39 years and another aged 40-49 years), and a reference group, aged between 20 and 34 years. Data concerning sociodemographic characteristics, obstetric history, previous morbid conditions, prenatal care and obstetric complications were gathered. The occurrence of potentially life-threatening conditions (PLTC-hemorrhagic, hypertensive, infectious and other complications), Maternal Near-Miss (MNM-clinical/laboratory criteria and management of severity) and Maternal Death (MD) was assessed, according to the WHO definition [8], in addition to data on pregnancy termination and perinatal results. The occurrence of delays in obstetric care was also assessed. For this purpose, specific questions in the questionnaire addressed delays for women seeking care, for reaching the health facility and also for obtaining the adequate and timely treatment for their complications. In addition specific rules for checking the occurrence of these delays were developed and systematically used for all cases.

Data was initially collected in a form following a one-year prospective surveillance by a research coordinator in each center. Subsequently, after the data was checked by the local investigator it was entered and stored in the platform OpenClinica® version 2.5.5 (Akaza Research, Waltham, MA, USA). Digital data was stored in a protected database from a computer accessible from the institutional website of the coordinating center of the study. Control of data quality was performed for every case in each participating center by using local rules and also in the coordinating center using general rules of completeness and consistency. Trained personnel from the coordinating center performed site monitoring visits for randomly selected cases (around 5% of those identified), cross-checking local data with the information already stored in the system. A manual of operation was developed and used by all of the centers involved in the study, in order to standardize concepts and data collection [18].

Sampling design corresponded to a single-stage cluster sampling, with 27 Primary Sampling Units (PSU), relative to the 27 centers (hospitals). The sampling design did not involve PSU stratification or data weighting because all centers were secondary or tertiary units with relatively similar teaching hospitals caring for at least 1000 deliveries per year. The unit of analysis was the medical record of each hospitalized woman with a PLTC (potentially life-threatening condition), MNM (maternal near-miss) or MD (maternal death). All variables studied had relatively low ICC values (intraclass correlation coefficients), demonstrating the necessary heterogeneity among study clusters. Specifically for the age variable, the ICC was 0.013 (95% CI 0.002-0.024) [19].

### Data analysis

Data analysis initially consisted of the distribution of women identified as PLTC, MNM and MD by age groups, obtaining prevalence ratios (PR) and respective confidence intervals (95% CI) adjusted by cluster effect of the study. Age groups were tested as a risk factor for the occurrence of MNM or MD. Information was not collected for women without maternal complications and therefore the distribution of live births for the study period according to maternal age categories was not possible. As a proxy, the number of live births (LB) was estimated according to the number of LB by maternal age group informed for the country in 2009 [20] so that health indicators related to maternal morbidity and mortality could be estimated: MNMR (maternal near-miss ratio per 100,000 LB), SMOR (severe maternal outcome ratio per 1,000 LB), MMR (maternal mortality ratio per 1,000 LB) and MNM:MD (the ratio between MNM and MD) [8]. Subsequently, the distributions of variables relative to socio demographic and clinical characteristics, obstetric history, habits, time and mode of pregnancy termination, neonatal results, and main causes of severe maternal morbidity by the three age groups currently analyzed. The differences between groups were evaluated by the χ^2^ test.

Finally, Poisson multiple regression analysis was used to identify factors that were independently and significantly associated with the worst outcomes (MD or MNM), compared to PLTC, for two models. The first model included only females aged 10 to 34 years (10-19 vs 20-34) for evaluating adolescent pregnant women, and the second model only women aged 20 to 49 years (20-34 vs 35-49) for the evaluation of older women. Women aged 20 to 34 years served as the reference group category in both models. The estimated coefficient, standard error (SE) of the coefficient, descriptive level (p) and prevalence ratio adjusted to cluster effect and remaining model variables, with their respective confidence interval (PR_adj_ [95% CI]) were obtained. For building the models for multiple analyses, all the predictive variables initially entered and then they were manually selected through the backward criteria in the Stata software v.7. Statistical analysis was carried out with software SPSS® version 17.0 (SPSS, Chicago, IL, USA) and Stata version 7.0 (StataCorp, College Station, Tx, USA). The descriptive level (α) was preset at 5% (95% confidence level) and a single-stage conglomerate sampling design was considered.

The study was previously approved by the Institutional Review Board (IRB of FCM/UNICAMP) of each center and by the National Research Committee prior to study initiation on May 5th 2009 (CEP 027/2009). This study did not require informed consent because data were collected exclusively from medical records immediately after patient discharge.

## Results

During the 12 months of the study, 9555 PLTC, MNM or MD cases were identified among 82,144 live births occurring in these hospitals during this period. Adolescents were responsible for 1713 (17.9%) of these cases, while older women aged 35 to 49 years accounted for a similar number of cases (1622-17%).

Table 1 shows a 1.25-fold higher risk of maternal near miss and maternal death for the 35-49 year age group (PR 1.25, 95% CI: 1.07-1.45), which was even higher in the 40-49 year age group (PR 1.52, 95% CI: 1.19-1.93). The prevalence ratio for near miss and maternal death was not higher for the total number of adolescents, although when ages 10 to 14 years were considered, the occurrence of 1 maternal death (MD) for every 2.3 MNN cases attracted attention. In the reference group, 1 death occurred (MD) for every 5.1 MNM cases. Table 1 also shows the MNMR and MMR values that increase with increasing age starting at the 15-19 year group, but with values also higher for the extreme adolescent group (girls aged 10-14 years).

**Table 1:** Distribution of women by age group and PLTC, MNM and MD

**Age (years)**	**%**	**PLTC**	**MNM**	**MD**	**PR (SMO: MNM + MD)**	**95% CI**	**ELB**	**MNMR/1000LB**	**SMOR/1000LB**	**MMR/100000 LB**	**MNM:MD**
10–19	17.9	18.2	15.2	15.72	0.89	0.77–1.04	16,388	7.14	8.48	134.2	5.3:1
10–14	1.2	1.2	0.9	2.13	0.97	0.57–1.65	797	8.78	12.55	376.4	2.3:1
15–19	16.8	17.0	14.3	13.19	0.88	0.76–1.03	15,591	7.06	8.27	121.9	5.8:1
20–34	65.1	65.3	62.6	67.95	1.00	Ref	57,435	8.39	10.05	165.4	5.1:1
35–49	17.0	16.5	22.2	16.23	*1.25*	*1.07–1.45*	8,321	20.55	23.31	276.4	7.4:1
35–39	12.1	11.8	14.8	10.7	1.19	1.00–1.41	6,506	17.52	19.83	230.6	7.6:1
40–49	4.9	4.7	7.4	5.7	*1.52*	*1.19–1.93*	1,815	31.40	35.81	440.8	7.1:1
**Total**	9,555	8,645	770	140			82,144	9.37	11.08	170.4	5.5:1

ELB: estimated live births; LB: number of live births; MD: Maternal Death; MMR: maternal mortality ratio; MNM: Maternal Near Miss; MNM:MD: maternal near miss to mortality ratio; MNMR: maternal near miss ratio; PLTC: Potentially Life Threatening Condition; SMOR: severe maternal outcome ratio; PR: prevalence ratio.

The number of ELB in each age group was obtained with the number of total live births in the period by age group distribution, according to data from SINASC (Brazil, 2009).

Maternal near miss ratio (MNMR), severe maternal outcome ratio (SMOR), maternal mortality ratio (MMR) and maternal near miss to mortality ratio (MNM:MD). Brazil, 2009-2010

Table 2 shows the difference between age groups regarding sociodemographic characteristics and clinical/obstetric history. Adolescents had a significantly higher proportion of non-whites, low school education, lack of a steady partner, low birthweight and nulliparity (only 12% of these reported having a previous pregnancy). In contrast, women over 35 years of age, compared to the reference group, had a higher proportion of low schooling, steady relationship, obesity, history of morbid clinical conditions (hypertension, diabetes, heart and thyroid disease), multiparity, history of previous abortions/cesarean sections and a lower number of prenatal visits.

**Table 2:** Distribution of women with any severe maternal morbidity by age group according to some characteristics, clinical and obstetric history

**Characteristics**	**10 – 19 years**	p*	**20 – 34 years**	p**	**35 – 49 years**
Ethnicity (non-white) (n)	60.8 (1,259)	*0.022*	56.9 (4,675)	0.912	56.7 (1,205)
Schooling (up to primary school) (n)	58.0 (1,275)	*<0.001*	41.6 (4,514)	*<0.001*	52.9 (1,134)
Marital status (No steady partner) (n)	62.3 (1,455)	*<0.001*	45.2 (5,255)	*<0.001*	36.6 (1,329)
BMI (n)	(630)	*<0.001*	(2,600)	*0.014*	(704)
Low weight	30.0		13.4		9.8
Adequate	33.8		27.1		24.9
Overweight	19.5		27.7		28.4
Obesity	16.7		31.8		36.9
Previous condition^&^ (n)	29.3 (1,446)	*<0.001*	49.6 (5,363)	*<0.001*	66.1 (1,432)
Hypertension	3.5	*<0.001*	16.7	*<0.001*	35.4
Diabetes mellitus	0.6	*<0.001*	2.2	*<0.001*	5.7
Cardiac	2.1	0.287	2.7	*<0.001*	4.7
Thyroid disease	0.6	*0.028*	1.3	*0.010*	2.8
Respiratory	3.1	0.458	2.8	0.776	2.7
Nephropathy	0.6	0.061	1.4	0.906	1.4
Thalassemia/sickle cell anemia	0.6	0.148	1.0	*0.012*	0.3
Collagenosis	0.4	0.539	0.6	0.508	0.5
Neoplasms	0.1	0.119	0.3	0.641	0.4
HIV/AIDS	0.3	*<0.001*	1.3	0.973	1.3
Low weight	1.0	*<0.001*	0.2	0.794	0.1
Obesity	12.9	*<0.001*	25.8	*0.033*	29.2
Smoking	3.9	*0.013*	5.9	0.599	6.4
Drug addiction	0.9	0.153	1.3	0.934	1.3
Other	3.4	0.100	4.9	*0.003*	8.3
Parity (n)	(1,707)	*<0.001*	(6,171)	*<0.001*	(1,615)
0	88.2		44.8		18.8
1–2	11.4		43.4		47.8
≥3	0.4		11.7		33.4
Previous abortion	7.3 (1,707)	*<0.001*	22.5 (6,169)	*<0.001*	37.6 (1,615)
Previous Cesarean section	5.6 (1,703)	*<0.001*	25.9 (6,084)	*<0.001*	36.8 (1,572)
Number of prenatal visits	(1,334)	*<0.001*	(4,863)	0.086	(1,260)
0	5.4		8.6		10.2
1–5	41.9		37.0		38.5
≥6	52.7		54.4		51.3

*p-values for proportions between adolescent and control groups.

**p-values for proportions between older age and control groups.

&previous conditions are not mutually exclusive.

Brazil, 2009-2010.

In general, adolescents had a better perinatal result (Table 3). These women had a significantly lower number of preterm deliveries, cesarean sections and low birth weight infants, and a higher number of vaginal deliveries, live births that were discharged in good conditions compared to the control group. In contrast, women over 35 years had a significantly higher number of cesarean sections and stillbirths than controls.

**Table 3:** Distribution of women with any severe maternal morbidity by age group according to time and mode of pregnancy termination and neonatal outcomes

**Outcomes**	**10 – 19 years**	p-value*	**20 – 34 years**	p-value**	**35 – 49 years**
Preterm birth <37 weeks (a)	36.5	*<0.001*	47.3	0.116	49.7
(n)	(1,510)		(5,444)		(1,381)
Mode or pregnancy termination (b)		*<0.001*		*0.037*	
Vaginal birth	27.8		21.7		19.6
Cesarean section	62.8		65.1		65.2
Abortion/ectopic	2.7		6.1		7.4
Still pregnant	6.7		7.0		7.8
(n)	(1,701)		(6,194)		(1,619)
Apgar 5th min <7 (c)	3.8	0.768	3.9	0.863	3.8
(n)	(1,430)		(4,954)		(1,241)
Birth weight <2.500 g (d)	32.4	*<0.001*	41.0	0.274	42.9
(n)	(1,468)		(5,147)		(1,308)
Neonatal condition at birth (e)		*0.014*		*0.019*	
Live birth	96.8		95.2		93.4
Stillbirth	3.2		4.7		6.6
(n)	(1,499)		(5,274)		(1,348)
Neonatal outcome (f)		*<0.001*		0.370	
Discharge	80.4		74.2		75.2
Admitted or transferred	17.3		23.0		22.7
Neonatal death	2.3		2.8		2.1
(n)	(1,395)		(4,839)		(1,212)

(a) 1220 cases excluded (still pregnant, abortion, ectopic pregnancy, or missing GA).

(b) 41 cases excluded (missed information on mode of pregnancy termination).

(c) 1930 cases excluded (still pregnant, abortion, ectopic pregnancy, fetal death, or missing Apgar).

(d) 1632 cases excluded (still pregnant, abortion, ectopic pregnancy, or missing weight).

(e) 1434 cases excluded (still pregnant, abortion, ectopic pregnancy, or missing condition at birth).

(f) 2109 cases excluded (still pregnant, abortion, ectopic pregnancy, or missing neonatal outcome).

*p-values for proportions between adolescent and control groups.

**p-values for proportions between older age and control groups.

Brazil, 2009-2010.

Inregards to the conditions identified as main causes of severe morbidity, the only differences found among the age groups were a lower proportion of hemorrhagic causes and a higher proportion of hypertensive causes among adolescent women. Furthermore, adolescents also had a higher proportion of situations identified as delays in obtaining adequate care for the complication (Table 4).

**Table 4:** Distribution of women with any severe maternal morbidity by age group according to main determining causes and delays in appropriate care

**Main causes of SMM**	**10 – 19 years**	p-value*	**20 – 34 years**	p-value**	**35 – 49 years**
Hemorrhage	19.4	*<0.002*	24.7	0.837	25.1
Hypertension	73.0	*0.009*	69.3	0.555	70.6
Infections	1.3	0.177	1.0	0.464	1.1
Clinical surgical conditions	11.0	0.898	10.9	0.457	9.9
(n)	(1,713)		(6,220)		(1,622)
Any delay (a)	57.7	*0,002*	52.7	0.503	53.9
(n)	(1550)		(5,686)		(1,480)

(a) 839 cases excluded (missing information on delays).

*p-values for proportions between adolescent and control groups.

**p-values for proportions between older age and control groups.

Brazil, 2009-2010.

The multivariate analyses model, including only adolescents and the reference group, showed that women with infectious, clinical/surgical and bleeding conditions (between three and five-fold) and who suffered any delay in care (two-fold) exhibited a higher risk of having SMO (MNM and MD). The risk was also higher when there were preexisting clinical conditions such as diabetes, neoplasm and others, in non-white skin color (between 1.5 and 2-fold). In contrast, obesity decreased by almost 50% the risk of MNM and MD. In this analysis, however, age was not identified as a factor associated with a worse case outcome (Table 5).

**Table 5:** Conditions independently associated with higher severity (SMO: MNM + MD) among women aged 10 to 19 compared to aged 20 to 34 years [n = 5,350]

**Variables**	**Coefficient**	**SE coeff.**	**p**	**PR**_**adj **_**[95% CI]**
Infectious conditions	1.66	0.33	*<0.001*	*5.23 [2.64–10.36]*
Clinical surgical conditions	1.52	0.16	*<0.001*	*4.58 [3.33–6.31]*
Hemorrhagic conditions	1.01	0.28	*<0.002*	*2.75 [1.54–4.89]*
Any delay	0.75	0.12	*<0.001*	*2.11 [1.64–2.72]*
Another previous condition	0.71	0.11	*<0.001*	*2.03 [1.60–2.57]*
Previous condition: diabetes	0.63	0.20	*0.003*	*1.88 [1.26–2.82]*
Previous condition: neoplasms	0.53	0.22	*0.024*	*1.71 [1.08–2.69]*
Ethnicity (nonwhite)	0.31	0.14	*0.041*	*1.36 [1.01–1.82]*
Previous condition: obesity	−0.65	0.17	*<0.002*	*0.52 [0.37–0.74]*
Constant	−3.62	0.21	*<0.001*	

PR_adj_: prevalence ratio adjusted by cluster effect and also by variables shown to be significant in the final model.

Poisson multiple regression, controlled by: Age 1 (10–19 years: 1/20–34: 0); Ethnicity (Nonwhite: 1/Other: 0); Schooling (up to primary school: 1/Other: 0); Marital status (no steady partner: 1/Other: 0); BMI (Low weight, adequate: 0/overweight, obesity: 1); Prenatal care at the same service (Absent: 1/Present: 0); Previous clinical conditions (Yes: 1/No: 0); Chronic hypertension (Yes: 1/No: 0); Obesity (Yes: 1/No: 0); Low weight (Yes: 1/No: 0); Diabetes mellitus (Yes: 1/No: 0); Smoking (Yes: 1/No: 0); Cardiac diseases (Yes: 1/No: 0); Respiratory diseases (Yes: 1/No: 0); Renal diseases (Yes: 1/No: 0); Thalassemia/sickle cell anemia (Yes: 1/No: 0); HIV/AIDS (Yes: 1/No: 0); Thyroid diseases (Yes: 1/No: 0); Neurologic diseases/epilepsy (Yes: 1/No: 0); Collagenosis (Yes: 1/No: 0); Neoplasms (Yes: 1/No: 0); Other preexisting condition (Yes: 1/No: 0); Drug addiction (Yes: 1/No: 0); Parity (0/≥1: 1); Previous abortion (Yes: 1/No: 0); Previous Cesarean section (Yes: 1/No: 0); Time since last delivery (years); Number of prenatal visits (up to 5: 0/≥6: 1); hemorrhagic conditions (Yes: 1/No: 0); Hypertensive conditions (Yes: 1/No: 0); Infectious conditions (Yes: 1/No: 0); Clinical surgical conditions (Yes: 1/No: 0); Any delay (Yes: 1/No: 0).

Brazil, 2009-2010.

The same analysis including only women aged 35 or older and the control group also showed a higher risk of having SMO (MNM or MD) due to infectious, clinical/surgical and bleeding conditions, and whether there was any delay in care (two to five-fold). Among the preexisting clinical conditions, kidney disease, diabetes, thalassemia/sickle cell anemia and others, in addition to drug addiction and having received prenatal care in another health service, were also greater risk factors for a worse outcome. It is also observed that previous obesity and lack of a steady partner were protective factors. Only in this analysis increased maternal age was shown to significantly increase by 25% the risk of a worse outcome for MNM or MD (Table 6).

**Table 6:** Conditions independently associated with higher severity (SMO: MNM + MD) among women aged 35 to 49 compared to women aged 20 to 34 years [n = 5,331]

**Variables**	**Coefficient**	**SE coeff.**	**p**	**PR**_**adj **_**[95% CI]**
Infectious conditions	1.55	0.26	*<0.001*	*4.71 [2.75–8.07]*
Clinical surgical conditions	1.37	0.19	*<0.001*	*3.94 [2.68–5.80]*
Hemorrhagic conditions	1.12	0.26	*<0.001*	*3.06 [1.81–5.18]*
Any delay	0.71	0.09	*<0.001*	*2.03 [1.70–2.42]*
Another previous condition	0.80	0.12	*<0.001*	*2.23 [1.75–2.84]*
Previous condition: renal diseases	0.78	0.22	*<0.002*	*2.17 [1.39–3.40]*
Previous condition: diabetes	0.62	0.17	*<0.002*	*1.87 [1.32–2.63]*
Thalassemia/sickle cell disease	0.78	0.26	*0.006*	*2.18 [1.28–3.70]*
Previous condition: drug addiction	0.40	0.12	*0.003*	*1.49 [1.15–1.92]*
Prenatal care in another service	0.25	0.09	*0.010*	*1.28 [1.07–1.53]*
Age (35–49 years × 20–34)	0.22	0.08	*0.007*	*1.25 [1.07–1.45]*
Previous condition: obesity	−0.52	0.14	*<0.002*	*0.59 [0.44–0.80]*
Marital status (no steady partner)	−0.65	0.14	*<0.001*	*0.52 [0.39–0.69]*
Constant	−3.51	0.22	*<0.001*	

PR_adj_: prevalence ratio adjusted by cluster effect and also by variables shown to be significant in the final model.

Multiple Poisson regression, controlled by: Age 2 (35–49 years: 1/ 20–34: 0); Ethnicity (Nonwhite: 1/Other: 0); Schooling (up to primary school: 1/Other: 0); Marital status (no steady partner: 1/Other: 0); BMI (Low weight, adequate: 0/overweight, obesity: 1); Prenatal care at the same service (Absent: 1/Present: 0); Previous clinical conditions (Yes: 1/No: 0); Chronic hypertension (Yes: 1/No: 0); Obesity (Yes: 1/No: 0); Low weight (Yes: 1/No: 0); Diabetes mellitus (Yes: 1/No: 0); Smoking (Yes: 1/No: 0); Cardiac diseases (Yes: 1/No: 0); Respiratory diseases (Yes: 1/No: 0); Renal diseases (Yes: 1/No: 0); Thalassemia/sickle cell anemia (Yes: 1/No: 0); HIV/AIDS (Yes: 1/No: 0); Thyroid diseases (Yes: 1/No: 0); Neurologic diseases/epilepsy (Yes: 1/No: 0); Collagenosis (Yes: 1/No: 0); Neoplasms (Yes: 1/No: 0); Other preexisting condition (Yes: 1/No: 0); Drug addiction (Yes: 1/No: 0); Parity (0/≥1: 1); Previous abortion (Yes: 1/No: 0); Previous Cesarean section (Yes: 1/No: 0); Time since last delivery (years); Number of prenatal visits (up to 5: 0/≥6: 1); hemorrhagic conditions (Yes: 1/No: 0); Hypertensive conditions (Yes: 1/No: 0); Infectious conditions (Yes: 1/No: 0); Clinical surgical conditions (Yes: 1/No: 0); Any delay (Yes: 1/No: 0).

Brazil, 2009-2010.

## Discussion and conclusions

It is well-known that maternal mortality risk is higher in two periods of age. The first is at the beginning of reproductive life or adolescence, going through a decrease and stabilization, and increasing again at the end of reproductive life [21]. The current analysis was carried out to assess factors that may be associated with a worse outcome of maternal complications and to evaluate whether the occurrence of NM also follows a pattern similar to that of maternal death regarding female age, in the context of a national surveillance study of severe maternal morbidity in Brazil.

The present study showed virtually the same proportion of cases with severe complications in both extremes of reproductive life (17%), in addition to almost 12% of repeat pregnancies in adolescence among those with SMM. As expected, the study also showed very specific differences between both extremes of age.

Among the total number of adolescents, the risk of SMO (NM or MD) was not significantly higher. However, in adolescents up to age 14, the extremely low case/fatality ratio captures attention. If adolescent pregnancy is already considered a problem [1], the problem is compounded especially in the 10-14 year age group [12]. All unfavorable conditions are exacerbated: physical, social, economic or violent incidents. Although the ratio of cases identified in this specific age group was small (1.2% of the total number of cases studied), not allowing a deeper analysis, a high risk was observed, reinforcing the need to expend sustained efforts to avoid pregnancy in this age period [12].

For older women, aged 35 years or more, the risk of unfavorable outcome was significantly higher than in the reference population. In the extreme age group (age > 40 years), this effect was even stronger. These findings have previously been reported by others [2,14,16,17]. Thus, as already known for maternal death [21], in the present study not only MMR, but also ratios of MNM increased swelled with increasing maternal age, except for the lower extreme of maternal age, again is in agreement with other published results [22-24]. The increased rate of MNM was probably the main finding of this analysis. According to the WHO [9], this indicator gives an estimate of the need to increment care and resources that are required to improve the results of obstetric care in certain regions. The differential gradient observed in different age groups could then also be used to prioritize investments and actions in groups identified as having a higher risk of MNM and MMR.

The comparison between several characteristics among the three maternal age groups of all women identified as having severe maternal morbidity in this study showed well-known differences among these groups, reflecting some unfavorable conditions for maternity in both extreme age groups, in comparison to an intermediate reproductive age. Thus, among adolescents with severe maternal morbidity, the non-white color was most frequent (poverty and greater vulnerability), lack of a steady partner and lower level of school education [1,12,13]. Adolescents may be at a disadvantage for not achieving the total number of school years and this inequality increases among non-white racial groups [25]. Similarly, nulliparity, vaginal delivery, low maternal weight and hypertension were more common as a cause of morbidity among adolescents. However, contrary to other studies [12], perinatal results were better in our cohort.

Obesity was significantly higher with increasing age. This finding confirms that the epidemic of obesity is the recent reality for the Brazilian population of reproductive aged women. Despite the lack of weight information for a large portion of the women in the study, more than 36% were above adequate weight even among adolescents; while 65% of women aged 35 years or older were overweight or obese. Obesity is not only a problem for the occurrence of SMM. It is also an issue that may possibly influence the future quality of life in these women. Nevertheless, obesity has also been previously identified as a risk factor for the occurrence of severe maternal morbidity [22,23]. The majority of preexisting morbid conditions were more prevalent among older women, especially hypertension, diabetes, heart and thyroid conditions. These conditions are more common with increasing age, and they have already been identified as risk factors for the occurrence of severe maternal morbidity [22].

Although a series of improvements have occurred in Brazil in economic, social and health terms in the last decades [26], maternal health is still characterized by relatively elevated mortality and morbidity rates. In addition to socioeconomic factors, our data highlights that extremely young and advanced maternal ages are associated with a higher risk of maternal complications that progress unfavorably to near-miss or maternal death. Special attention should be devoted to the provision of health services both for prenatal care and management during delivery and postpartum period.

## Abbreviations

CI: Confidence intervals; ELB: Estimated number of live births; ICC: Intraclass correlation coefficients; LB: Live births; MD: Maternal death; MMR: Maternal mortality ratio; MNM: Maternal near miss; MNM:MD: The ratio between MNM and MD; MNMR: Maternal near-miss ratio; NM: Near miss; PLTC: Potentially life-threatening conditions; PR: Prevalence ratio; PSUm: Primary Sampling Units; SE: Standard error; SMM: Severe maternal morbidity; SMOR: Severe maternal outcome ratio; WHO: World Health Organization.

## Competing interests

All authors declare that they have no competing interests.

## Authors’ contributions

The idea for the study and this specific analytical approach arose in a group discussion among all the authors. Analyses were planned and performed by FCO, FGS, JLPS, JGC and MHS. The first version of the manuscript was drafted by FCO, and then complemented with suggestions from all the others, and mainly FGC, JLPS, JGC, and RCP. All authors contributed to the development of the study protocol, read and approved the final version of the manuscript.

## Pre-publication history

The pre-publication history for this paper can be accessed here:

http://www.biomedcentral.com/1471-2393/14/77/prepub

## Brazilian Network for the Surveillance of Severe Maternal Morbidity Group

Rodrigo S. Camargo, Vilma Zotareli, Lúcio T. Gurgel, Eliana M Amaral, Lale Say, Robert C Pattinson, Marilza V Rudge, Iracema M Calderon, Maria V Bahamondes, Danielly S Santana, Simone P Gonçalves, Olímpio B Moraes Filho, Simone A Carvalho, Francisco E Feitosa, George N Chaves, Ione R Brum, Gloria C Saint’Ynes, Carlos A Menezes, Patricia N Santos, Everardo M Guanabara, Elson J Almeida Jr, Joaquim L Moreira, Maria R Sousa, Frederico A Peret, Liv B Paula, Luiza E Schmaltz, Cleire Pessoni, Leila Katz, Adriana Bione, Antonio C Barbosa Lima, Edilberto A Rocha Filho, Melania M Amorim, Debora Leite, Ivelyne Radaci, Marilia G Martins, Frederico Barroso, Denis J Nascimento, Cláudio S Paiva, Moises D Lima, Djacyr M Freire, Roger D Rohloff, Simone M Rodrigues, Sergio M Costa, Lucia C Pfitscher, Adriana G Luz, Daniela Guimaraes, Gustavo Lobato, Marcos Nakamura-Pereira, Eduardo Cordioli, Alessandra Peterossi, Cynthia D Perez, Jose C Peraçoli, Roberto A Costa, Nelson L Maia Filho, Jacinta P Matias, Silvana M Quintana, Elaine C Moises, Fátima A Lotufo, Luiz E Carvalho, Carla B Andreucci, Elvira Zanetti, Márcia M Aquino, Maria H Ohnuma, Rosiane Mattar and Felipe F Campanharo.
